# Routes to Diagnosis and Stage at Diagnosis of Colorectal Cancer in Patients Aged ≥ 50 Years: A Multicentre Retrospective Cohort Study From North Wales, UK

**DOI:** 10.7759/cureus.118400

**Published:** 2026-10-22

**Authors:** Mikaella Toquero, Sushmitha Thiagarajah Naidu, Anna D Williams, Joseph Waddon, Rebekah Emmas, Aashna Das, Asifa Noor, Vignesh Balasubaramaniam, Steve Dixon

**Affiliations:** 1 Hospital Medicine, Wrexham Maelor Hospital, Wrexham, GBR; 2 Surgery, Countess of Chester Hospital, Chester, GBR; 3 Surgery, The Grange University Hospital, Llanyrafon, GBR; 4 General Surgery, Wrexham Maelor Hospital, Wrexham, GBR

**Keywords:** colorectal cancer (crc), colorectal cancer (crc) screening, colorectal cancer staging, diagnostic pathway, faecal immunochemical test, fit, late-onset colorectal cancer, north wales, route to diagnosis, stage at diagnosis

## Abstract

‘Route to diagnosis’, the clinical pathway through which a cancer is first identified, is associated with stage at presentation in colorectal cancer (CRC), but recent regional data in the UK describing this relationship following the wider adoption of symptomatic faecal immunochemical testing (FIT) is limited. This study describes contemporary routes to diagnosis among patients aged ≥ 50 years with CRC in a single Welsh health board and compares unadjusted stage distribution between routes.

A retrospective cohort study was conducted across three secondary care sites within Betsi Cadwaladr University Health Board, North Wales, including patients aged ≥ 50 years with newly diagnosed primary colorectal cancer between 1 January 2023 and 31 December 2024. Routes were Bowel Screening Wales (BSW), asymptomatic FIT, symptomatic FIT, symptoms only, emergency presentation, and incidental detection. Stage was the pre-treatment tumour, node, and metastasis (TNM) stage where available; otherwise, pathological stage. Stage was compared across routes using Pearson’s chi-square test, with four pre-specified pairwise comparisons of advanced-stage (III-IV) disease. The asymptomatic FIT group was described only.

Of 976 patients, two (0.2%) were diagnosed via asymptomatic FIT. Among the remaining 974, symptomatic FIT was the most common route (506, 52.0%), followed by BSW (172, 17.7%), symptoms only (131, 13.4%), emergency presentation (112, 11.5%), and incidental detection (53, 5.4%). Overall, 515/974 (52.9%) had advanced-stage disease, and stage differed by route (p < 0.001). Advanced disease was lowest in BSW (37.8%), intermediate in symptomatic FIT (51.8%), higher in symptoms-only (60.3%) and emergency (60.7%) diagnoses, and highest in incidental diagnoses (77.4%). Compared with BSW, the proportion with advanced disease was 22.9 percentage points higher in emergency presentations (95% CI 11.3-34.5) and 39.6 points higher in incidental diagnoses (95% CI 26.2-53.0). The difference between symptomatic FIT and emergency presentations (8.9 points, 95% CI −1.1 to 19.0) was not statistically significant.

In this unadjusted analysis, which may partly reflect differences in age, tumour location and staging method, screening-detected cancers had the lowest proportion of advanced disease and incidental diagnoses the highest. Just over half of cancers diagnosed after a positive symptomatic FIT were stage III-IV; as only diagnosed patients were included, this does not assess FIT effectiveness. These findings provide a regional benchmark and identify pathways where the reasons for late-stage diagnosis warrant further investigation.

## Introduction

Colorectal cancer (CRC) may be diagnosed through several distinct pathways. In this study, the ‘route to diagnosis’ is the clinical pathway through which the cancer was first identified: population screening, symptomatic referral, with or without a faecal immunochemical test (FIT), emergency presentation, or incidental detection - this is defined in the methods section. These routes are associated with different stage distributions at diagnosis, with screening-detected cancers generally identified at an earlier stage and emergency presentations more frequently associated with advanced disease and complications such as obstruction or perforation [1,2]. Symptomatic and incidental routes are more heterogeneous, reflecting variation in symptom recognition, referral patterns and the circumstances in which the cancer is identified [3,4]. 

Stage at diagnosis remains a major determinant of treatment options and prognosis. In recent years, faecal immunochemical testing (FIT) has become established within UK primary care pathways for patients with lower gastrointestinal symptoms, supporting risk stratification and prioritisation for further investigation [5-9]. In the UK, FIT is a triage test rather than a diagnostic standard in which a positive result or high-risk symptoms prompts referral for colonoscopy (or CT colonography where colonoscopy is unsuitable), which remains the definitive investigation [5,7]. FIT therefore determines who is prioritised for endoscopy rather than establishing the diagnosis itself. However, symptomatic FIT does not replace other diagnostic routes and colorectal cancers continue to be diagnosed through screening, emergency admission, symptom-based referral without FIT and incidental investigation. 

Existing evidence in the UK consistently demonstrates an association between route to diagnosis and stage, but much of this literature predates the wider adoption of symptomatic FIT or is derived from large national datasets, particularly from England [1,2,10-15]. Few studies in contemporary practice have distinguished between symptomatic patients referred following FIT testing and those referred based on symptoms alone, or between incidental and emergency diagnoses. In particular, incidental diagnoses remain underexplored. National datasets are valuable for identifying broad population trends but may obscure local variation in screening uptake, referral practice and service configuration. Recent qualitative work from Wales has also highlighted patient and caregiver concerns regarding the diagnostic journey, supporting the value of locally grounded evaluation of current pathways [16]. 

The primary objective of this study was to compare the stage distribution at diagnosis across diagnostic routes among patients aged ≥ 50 years in North Wales between January 2023 and December 2024. Secondary objectives were to describe presenting symptoms, tumour location and clinical characteristics by route. Patients aged ≥ 50 years were studied because this is the conventional definition of late-onset CRC and corresponds to the age range of Bowel Screening Wales (BSW), whose lower age limit has been reduced in phases towards 50 years over the last few years [17]; younger patients are the subject of separate work. The study is a descriptive audit of current pathways. It was not designed to evaluate the effect of introducing FIT, as no historical comparator was available and the comparisons between routes are unadjusted.

## Materials and methods

Study design and setting 

This was a retrospective multicentre cohort study conducted as a clinical audit across Betsi Cadwaladr University Health Board (BCUHB), North Wales. Patients with newly diagnosed colorectal cancer between 1 January 2023 and 31 December 2024 were identified from three secondary care sites: Wrexham Maelor Hospital, Glan Clwyd Hospital and Ysbyty Gwynedd. The initial cohort was identified from the colorectal cancer multidisciplinary team (MDT) database, maintained by the MDT coordinator, which records all patients discussed at the colorectal MDT across the three sites. BCUHB is the single NHS health board serving North Wales; the three study sites are district general hospitals operating within the same health board and shared referral system. Although BCUHB has university affiliations and an established research infrastructure, the present study is based on routine clinical audit data from secondary care hospital settings. The study was designed to provide a descriptive assessment of contemporary routes to diagnosis, presenting features and stage distribution. This cohort represents a defined subset of a wider BCUHB colorectal cancer dataset, restricted to patients meeting the age criterion and with sufficient route-to-diagnosis and staging data available for analysis. ‘Sufficient data' meant a documented route to diagnosis and a determinable stage.

Inclusion and exclusion criteria 

Table 1 shows the inclusion and exclusion criteria of the study. Patients were eligible for inclusion if they were aged ≥ 50 years at diagnosis, had a newly diagnosed primary cancer of the colon or rectum, and were diagnosed at one of the three North Wales secondary care sites within the study period. ‘Newly diagnosed’ was defined as the first diagnosis of primary colorectal cancer made within the study period. Patients were excluded if they were under 50 years of age at diagnosis, if their tumour originated from the appendix, anus, or an unknown primary site, if they had a prior history of colorectal cancer, or if no stage could be determined. Figure 1 shows patient selection for this study, including the reasons for exclusion. Some patients met more than one exclusion criterion, so the number excluded for each reason is not reported.

**Table 1:** Inclusion and exclusion criteria of the study Exclusion criteria were not mutually exclusive; some patients met more than one.

Inclusion Criteria	Exclusion Criteria
Aged ≥ 50 years at diagnosis	Aged < 50 years at diagnosis
Newly diagnosed primary cancer of the colon or rectum	Tumour originated from appendix, anus, or an unknown primary site
One of the North Wales secondary sites, which are Wrexham Maelor Hospital, Glan Clwyd Hospital, and Ysbyty Gwynedd	Previous history of colorectal cancer
Patient from the study period from 1 January 2023 to 31 December 2024	No stage could be determined

**Figure 1:**
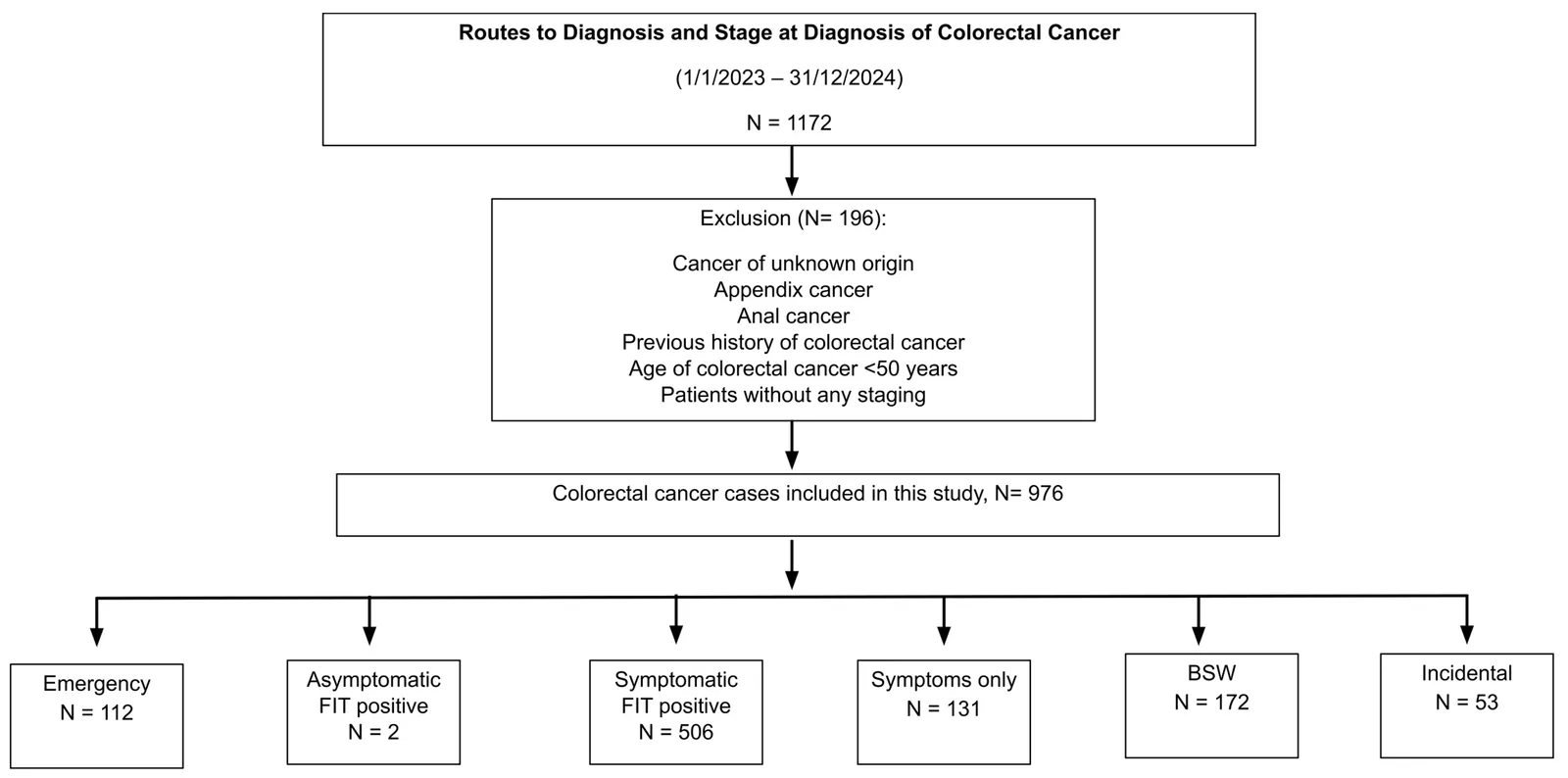
Flow chart illustrating patient selection for this study FIT: faecal immunochemical test; BSW: Bowel Screening Wales; N: number of patients.

Ethical considerations

This study was a retrospective review of routinely collected, anonymised clinical data, registered and conducted as a clinical audit within BCUHB (audit registration number: 2733). Formal research ethics committee approval and individual patient consent were not required. The study was conducted in line with the principles of the Declaration of Helsinki. Data were extracted by members of the clinical team with existing access to the records and entered into the study spreadsheet without any identifiable information. This information was stored on password-protected BCUHB network storage.

Data sources

Data were extracted from two electronic systems used across BCUHB: the Welsh Clinical Portal (WCP) and Epro for clinic letters. These sources were used to identify the route of diagnosis, such as screening through BSW, symptomatic or asymptomatic FIT referral, symptoms-only referral, emergency presentation, or incidental detection. They also provided demographic and clinical data such as presenting symptoms and comorbidities. Investigation results included FIT values, haemoglobin levels, and radiological and pathological staging. Both systems were reviewed for every patient. Clinical correspondence and multidisciplinary team records were reviewed to verify route classification and staging where required. Where the route to diagnosis was unclear from a single source, the wider clinical record was reviewed to assign the most appropriate category. Data were collected by eight reviewers using a common data dictionary; where sources conflicted, the multidisciplinary team (MDT) outcome record took precedence. Treatment data were recorded where relevant to interpret the stage at diagnosis. 

Variables and definitions

Demographic variables recorded included age at diagnosis and sex. Comorbidity status was recorded as a binary variable (yes/no), with 'yes' assigned where a patient had two or more pre-existing diagnosed medical conditions prior to CRC diagnosis. Smoking status was recorded as 'yes' if the patient had been a current or former smoker and recorded as 'no' if they had never smoked. CRC-related symptoms were defined as rectal bleeding (PR), change in bowel habit, abdominal pain, weight loss or loss of appetite, and anaemia. Tumour location within the colon or rectum was also recorded and divided into right colon, transverse colon, left colon and rectum according to the documented primary tumour site. The right colon included caecum, ascending colon and hepatic flexure. The left colon included splenic flexure, descending colon, sigmoid and recto-sigmoid. 

BSW included cancers identified through the national bowel screening programme. Symptomatic FIT included patients investigated following a positive quantitative FIT requested because of CRC-related symptoms; a positive FIT was defined as > 10 µg Hb/g faeces. Asymptomatic FIT included patients with a positive FIT but no documented CRC-related symptoms at the time of testing. Symptoms-only included patients referred from primary care because of CRC-related symptoms who had a negative FIT result or no available FIT result. In the absence of recorded evidence, FIT testing could not be confirmed; therefore, classification was based on the information available in the clinical record. Emergency presentations included patients diagnosed following an unplanned hospital admission requiring urgent assessment and treatment. Patients already investigated in the community (e.g. with FIT) were classified as emergency presentations if an acute hospital presentation prompted diagnostic investigation. Meanwhile, incidental diagnosis included cancers identified during investigation or treatment of an unrelated condition in the absence of a prior suspected-cancer referral, in a non-emergency setting.

Staging at diagnosis was recorded according to the tumour, node and metastasis (TNM) eighth edition staging system; this was the edition available during the timeframe of the data collected. Where available, clinical/radiological (pre-treatment) staging was used; where pre-treatment staging was unavailable, pathological (post-treatment) staging was recorded. Pre-treatment staging was unavailable in most patients presenting as an emergency, where colorectal cancer was identified only at the time of urgent surgical intervention and complete staging was subsequently done after surgical intervention. Pre-treatment staging was used for 892 patients (91.4%), and pathological staging was used for 84 patients (8.6%). No pathological stage was recorded after neoadjuvant therapy. Because the staging method differs systematically between routes, this may influence comparisons (see limitations).

Data were entered into a structured spreadsheet using a predefined data dictionary to standardise variable coding across the three sites. Data cleaning was performed prior to analysis, including removal of duplicate entries, verification of discrepancies against source clinical records and standardisation of categorical variables. Missing data was assessed for each variable and reported with analysed denominators; no imputation was performed.

Statistical analysis

Statistical analysis was performed using Jamovi, version 2.7 (The jamovi Project, Sydney, Australia). Descriptive statistics were used to summarise the frequency of each route to diagnosis and the distribution of stage at diagnosis across the cohort. The association between route of diagnosis and stage at diagnosis was assessed using Pearson’s chi-square test. The two asymptomatic FIT patients were described separately and excluded specifically from statistical comparisons (N = 974), but included overall.

Categorical variables were compared using Pearson’s chi-square test. Patients with missing smoking or comorbidity data were excluded from those analyses, and missing numbers are reported separately. Age was reported descriptively. Effect sizes are reported as Cramér’s V. Expected cell counts were checked for each test; fewer than 20% were below five in all analyses, so Pearson’s chi-square test was appropriate.

Advanced-stage disease was defined as stage III or IV. Four clinically relevant pairwise comparisons of advanced-stage disease were pre-specified and undertaken following the overall comparison across diagnostic routes: BSW vs emergency, BSW vs incidental, symptomatic FIT vs emergency and symptomatic FIT vs incidental. These comparisons were chosen to contrast the two pathways most associated with earlier diagnosis (BSW and symptomatic FIT) with the two most associated with late presentation (emergency and incidental), as identified in the existing literature, and were limited to four to reduce multiple testing. Pearson's chi-square test was used for these comparisons, and the absolute difference in the proportion with advanced-stage disease was reported with a 95% confidence interval. 

As this was a descriptive study, all comparisons were unadjusted, with no correction for multiple testing or for factors such as age or tumour site. A p-value < 0.05 was considered statistically significant. 

Artificial intelligence disclosure

Artificial intelligence (AI) was used mainly to improve language, clarity and organisation of the manuscript. There was limited use to help refine the discussion and interpretation. All AI-assisted content was reviewed and checked by the authors, who remain responsible for the accuracy and integrity of the manuscript.

## Results

Figure 1 shows patient selection for this study. Of 1,172 patients identified with CRC during the study period, 196 were excluded, and 976 patients met the inclusion criteria for final analysis. Of the 976 patients included, two were diagnosed following an asymptomatic positive FIT. Given the small size of this group, these patients are described only and excluded from Table 2, which summarises the baseline characteristics of the remaining 974 patients. The most common route to diagnosis was symptomatic FIT (506/974, 52.0%), followed by BSW (172/974, 17.7%), symptoms only (131/974, 13.4%), emergency presentation (112/974, 11.5%) and incidental detection (53/974, 5.4%).

**Table 2:** Baseline characteristics by diagnostic route FIT: faecal immunochemical test; BSW: Bowel Screening Wales; SD: standard deviation; n: number of patients; N: total number of patients; χ²: chi-square statistic; df: degrees of freedom. Cramér’s V indicates the effect size. Age is presented as mean ± standard deviation in years. Percentages for comorbidity and smoking are of patients with known status; missing data are shown separately and were excluded from the tests. P-values were calculated using Pearson’s chi-square test. Asymptomatic FIT-positive patients (n = 2) are not shown.

Variable	Emergency n (%)		Symptomatic +ve FIT n (%)	Symptoms only n (%)	BSW n (%)	Incidental n (%)	p-value		χ²	df	Cramér’s V
Total patients N = 974	112 (11.5%)		506 (52.0%)	131 (13.4%)	172 (17.7%)	53 (5.4%)	-				
Age, mean (± SD)	73.5 (±10.9)		73.6 (±10.1)	73.8 (±10.4)	64.1 (±7.1)	75.6 (±9.7)	-				
Male	69 (61.6%)		298 (58.9%)	71 (54.2%)	104 (60.5%)	30 (56.6%)	0.771		1.81	4	0.043
Female	43 (38.4%)		208 (41.1%)	60 (45.8%)	68 (39.5%)	23 (43.4%)					
Tumour Location											
Right colon	51 (45.5%)		161 (31.8%)	50 (38.2%)	41 (23.8%)	23 (43.4%)	< 0.001		59.59	12	0.143
Transverse colon	8 (7.1%)		20 (4.0%)	6 (4.6%)	12 (7.0%)	3 (5.7%)					
Left colon	44 (39.3%)		167 (33.0%)	28 (21.4%)	83 (48.3%)	18 (34.0%)					
Rectum	9 (8.0%)		158 (31.2%)	47 (35.9%)	36 (20.9%)	9 (17.0%)					
Comorbidities											
Comorbidities ≥ 2 (if present)	67 (62.0%)		306 (61.4%)	77 (60.2%)	77 (45.6%)	34 (65.4%)	0.004		15.15	4	0.126
Missing data	4		8	3	3	1	-				
Smoking											
Smoking status (current or former smokers)	52 (48.1%)		255 (50.7%)	69 (53.9%)	74 (46.0%)	28 (57.1%)	0.554		3.03	4	0.056
Missing data	4		3	3	11	4	-				

Mean age at diagnosis was lowest in the BSW group (64.1 ± 7.1 years) and highest in the incidental group (75.6 ± 9.7 years), with the remaining routes ranging from 73.5 to 73.8 years. The two asymptomatic FIT patients had a mean age of 64.0 years. Sex distribution did not differ significantly by route (p = 0.771), but tumour location did (p < 0.001), with rectal tumours proportionally more common among symptomatic FIT (158/506, 31.2%) and symptoms-only (47/131, 35.9%) patients than among those diagnosed via BSW (36/172, 20.9%) or emergency presentation (9/112, 8.0%). Comorbidity status was missing for 19 patients and smoking status for 25; percentages for these variables are based on patients with known status. Comorbidity differed significantly by route (p = 0.004), being lowest among BSW-detected patients (77/169, 45.6%) and highest among incidental diagnoses (34/52, 65.4%). Smoking status did not differ significantly by route (p = 0.554); the proportion of current or former smokers was numerically lowest among BSW-detected patients (74/161, 46.0%) and highest among incidental diagnoses (28/49, 57.1%).

Table 3 presents recorded presenting symptoms among patients diagnosed through non-emergency routes, excluding asymptomatic FIT patients. Symptom recording differed significantly by route for all five symptom categories (all p < 0.001). Symptomatic FIT and symptoms-only patients had similar profiles, with change in bowel habit the most common presenting symptom in both groups (273/506, 54.0% and 61/131, 46.6%). In symptomatic FIT patients, this was followed by anaemia (217/506, 42.9%), rectal bleeding (197/506, 38.9%), abdominal pain (140/506, 27.7%) and weight loss and/or loss of appetite (116/506, 22.9%). In symptoms-only patients, it was followed by rectal bleeding (57/131, 43.5%), anaemia (55/131, 42.0%), weight loss and/or loss of appetite (33/131, 25.2%), and abdominal pain (31/131, 23.7%). Symptoms were recorded less often in incidental patients (2/53 to 12/53, 3.8-22.6%), among whom anaemia was most common (12/53, 22.6%), followed by weight loss and/or loss of appetite (7/53, 13.2%). Symptoms were rarely recorded in BSW-detected patients (1/172 to 10/172, 0.6-5.8%), of whom anaemia was the most common (10/172, 5.8%).

**Table 3:** Recorded presenting symptoms by diagnostic route among patients diagnosed through non-emergency routes, excluding asymptomatic FIT positive patients FIT: faecal immunochemical test; BSW: Bowel Screening Wales; n: number of patients; N: total number of patients; χ²: chi-square statistic; df: degrees of freedom. Cramér’s V indicates the effect size. Values are presented as the number of patients with the symptom/total number in each diagnostic route (%). Percentages were calculated within each diagnostic-route group. P-values were calculated using Pearson’s chi-square test to compare the presence or absence of each symptom across the four diagnostic routes.

Presenting symptom, n (%)	Symptomatic FIT positive, n (%)	Symptoms only, n (%)	BSW, n (%)	Incidental, n (%)	p-value*	χ²	df	Cramér’s V
Rectal bleeding	197/506 (38.9%)	57/131 (43.5%)	5/172 (2.9%)	3/53 (5.7%)	< 0.001	104.84	3	0.349
Change in bowel habit	273/506 (54.0%)	61/131 (46.6%)	5/172 (2.9%)	3/53 (5.7%)	< 0.001	168.47	3	0.442
Abdominal pain	140/506 (27.7%)	31/131 (23.7%)	2/172 (1.2%)	2/53 (3.8%)	< 0.001	65.77	3	0.276
Weight loss and/or loss of appetite	116/506 (22.9%)	33/131 (25.2%)	1/172 (0.6%)	7/53 (13.2%)	< 0.001	48.61	3	0.237
Anaemia	217/506 (42.9%)	55/131 (42.0%)	10/172 (5.8%)	12/53 (22.6%)	< 0.001	85.33	3	0.315

The subtypes of emergency presentation are presented in Table 4. Other acute presentations (abdominal pain, loss of appetite, vomiting) were most common (51/112, 45.5%), followed by obstruction (42/112, 37.5%), unrelated findings on imaging during emergency admission (13/112, 11.6%) and perforation (6/112, 5.4%). Obstruction or perforation therefore accounted for 48/112 (42.9%) of emergency presentations. 

**Table 4:** Subtypes of emergency presentation n - number of patients. Percentages were calculated using all 112 emergency presentations; each patient is counted in one category only.

Emergency presentation subtype	Patients, n (%)
Obstruction	42/112 (37.5%)
Perforation	6/112 (5.4%)
Unrelated finding on imaging during emergency admission	13/112 (11.6%)
Other acute presentations (abdominal pain, loss of appetite, vomiting)	51/112 (45.5%)

Stage by diagnostic route is shown in Table 5. Excluding the two asymptomatic FIT patients, stage distribution differed significantly across the five main routes (p < 0.001). Across these 974 patients, stage III was most common (339/974, 34.8%), followed by stage I (243/974, 24.9%), stage II (197/974, 20.2%), stage IV (176/974, 18.1%) and stage 0 (19/974, 2.0%). Stage I was most frequent in BSW-detected patients (76/172, 44.2%), who also had the lowest proportion of stage IV disease (14/172, 8.1%). Stage III was the most common in emergency (42/112, 37.5%), symptomatic FIT (176/506, 34.8%), and symptoms-only (52/131, 39.7%) diagnoses, whereas stage IV was the most common stage in incidental diagnoses (23/53, 43.4%). These comparisons are unadjusted, and pre-treatment staging was less often available for emergency presentations. Both asymptomatic FIT patients had early-stage disease (stage 0 and stage II) and are described only.

**Table 5:** Stage by diagnostic route FIT: faecal immunochemical test; BSW: Bowel Screening Wales; n: number of patients; N: total number of patients; χ²: chi-square statistic; df: degrees of freedom. Cramér’s V indicates the effect size. Percentages were calculated within each diagnostic-route group. P-value represents the overall comparison of stage distribution across diagnostic routes using Pearson’s chi-square test. Asymptomatic FIT-positive patients (n = 2) are not shown.

Stage	Emergency n (%)	Symptomatic +ve FIT n (%)	Symptoms only n (%)	BSW n (%)	Incidental n (%)	Total, N	p-value	χ²	df	Cramér’s V
Stage 0	0 (0.0%)	11 (2.2%)	0 (0.0%)	7 (4.1%)	1 (1.9%)	19	< 0.001	101.27	16	0.161
Stage I	8 (7.1%)	127 (25.1%)	27 (20.6%)	76 (44.2%)	5 (9.4%)	243				
Stage II	36 (32.1%)	106 (20.9%)	25 (19.1%)	24 (14.0%)	6 (11.3%)	197				
Stage III	42 (37.5%)	176 (34.8%)	52 (39.7%)	51 (29.7%)	18 (34.0%)	339				
Stage IV	26 (23.2%)	86 (17.0%)	27 (20.6%)	14 (8.1%)	23 (43.4%)	176				
Total, N	112	506	131	172	53	974	-			

Table 6 shows that, among the 974 patients in the five main routes, 459 (47.1%) had early-stage (0-II) disease, and 515 (52.9%) had advanced-stage (III-IV) disease. The proportion with advanced disease was highest in incidental diagnoses (41/53, 77.4%), followed by emergency (68/112, 60.7%) and symptoms-only (79/131, 60.3%) diagnoses. It was intermediate in symptomatic FIT (262/506, 51.8%) and lowest in BSW (65/172, 37.8%). These are unadjusted proportions. Both asymptomatic FIT patients had early-stage disease and are described only.

**Table 6:** Early versus advanced best available TNM stage by diagnostic route FIT: faecal immunochemical test; BSW: Bowel Screening Wales; TNM: tumour, node, and metastasis; n: number of patients. Early-stage disease was defined as stages 0–II and advanced-stage disease as stages III–IV. Percentages were calculated within each diagnostic-route group. Asymptomatic FIT-positive patients (n = 2) are not shown.

Diagnostic route	Early (Stage 0-II), n (%)	Advanced (Stage III-IV), n (%)
Emergency	44 (39.3%)	68 (60.7%)
Symptomatic +ve FIT	244 (48.2%)	262 (51.8%)
Symptoms only	52 (39.7%)	79 (60.3%)
BSW	107 (62.2%)	65 (37.8%)
Incidental	12 (22.6%)	41 (77.4%)
Total	459 (47.1%)	515 (52.9%)

Table 7 shows four pre-specified pairwise comparisons of advanced-stage disease, with absolute differences and 95% confidence intervals. BSW-detected patients were significantly less likely to have advanced disease than emergency patients (37.8% vs 60.7%; p < 0.001) or incidental patients (37.8% vs 77.4%; p < 0.001). The proportion with advanced disease was also significantly lower in symptomatic FIT patients than in incidental patients (51.8% vs 77.4%; p < 0.001). The difference between symptomatic FIT and emergency patients (51.8% vs 60.7%; p = 0.086) was not statistically significant, but this should not be taken to mean the two routes are equivalent. These comparisons are unadjusted for age, sex, tumour location, and comorbidity.

**Table 7:** Pairwise comparisons of advanced-stage colorectal cancer by diagnostic pathway FIT: faecal immunochemical test; BSW: Bowel Screening Wales; n: number of patients with advanced-stage disease; χ²: chi-square statistic; df: degrees of freedom. Cramér’s V indicates the effect size. P-values were calculated using Pearson’s chi-square test, with no continuity correction or adjustment for multiple comparisons.

Route A	Route B	Advanced stage in Route A, n (%)	Advanced stage in Route B, n (%)	p-value	χ²	df	Cramér’s V	Difference in advanced stage, % points (95% CI)
BSW	Emergency	65/172 (37.8%)	68/112 (60.7%)	< 0.001	14.32	1	0.225	22.9 (11.3 to 34.5)
BSW	Incidental	65/172 (37.8%)	41/53 (77.4%)	< 0.001	25.46	1	0.336	39.6 (26.2 to 53.0)
Symptomatic +ve FIT	Emergency	262/506 (51.8%)	68/112 (60.7%)	0.086	2.94	1	0.069	8.9 (-1.1 to 19.0)
Symptomatic +ve FIT	Incidental	262/506 (51.8%)	41/53 (77.4%)	< 0.001	12.65	1	0.15	25.6 (13.5 to 37.7)

## Discussion

The findings of this study indicate that the route of diagnosis is associated with stage at presentation in this cohort. Patients diagnosed through BSW were significantly less likely to have advanced-stage disease than those diagnosed via emergency admission or incidentally. Symptomatic FIT accounted for the largest proportion of diagnoses (506/974, 52.0%), and just over half of patients diagnosed through this pathway had stage III-IV disease (262/506, 51.8%). Because this cohort comprises only patients already diagnosed with CRC, this is a characteristic of the cohort and not an assessment of FIT effectiveness or sensitivity; this study cannot show whether symptomatic FIT improves earlier detection. These are unadjusted associations. The pairwise differences are consistent with the wider UK evidence base linking route of diagnosis to stage [1,2,15].

The markedly younger mean age in the BSW group (64.1 years) compared with the other routes (73.5-75.6 years) largely reflects eligibility for screening. During the study period, BSW invited people up to the age of 74, and its lower age limit was reduced in phases towards 50 years [17]. Patients outside this range could not be screened during the study period. Screening-detected patients also had a significantly lower prevalence of comorbidity than other routes (77/169, 45.6% vs 60.2-65.4%; p = 0.004), whereas smoking history did not differ significantly by route. Age restriction, comorbidity, participation in screening and tumour characteristics are plausible contributors to the stage differences, but their individual effects were not assessed. A healthy-participant effect (people who take up screening tend to be healthier than those who do not) and length-time bias (screening preferentially detects slower-growing tumours that remain detectable for longer) may also contribute to the favourable stage distribution in this group, but these are hypotheses that these data cannot test.

The findings in the incidental group warrant particular attention. Although relatively small in number, 41/53 (77.4%) of patients diagnosed incidentally had stage III-IV disease, and 23/53 (43.4%) had stage IV disease, the highest proportions among the diagnostic routes examined. Incidental diagnosis should not be equated with the absence of symptoms - anaemia was recorded in 12/53 (22.6%) and weight loss or loss of appetite in 7/53 (13.2%) of these patients. This suggests that some had symptoms that had not prompted colorectal investigation, while others may have had clinically occult disease; these explanations require further study. Cancers found during emergency admissions were classified as emergency presentations, so the two groups do not overlap, although misclassification at the boundary cannot be excluded. The incidental group also had the highest prevalence of comorbidity (34/52, 65.4%). Patients with more pre-existing conditions are more likely to undergo investigations for reasons unrelated to CRC, increasing the opportunity for an incidental finding [18]. Incidental diagnosis has received less attention in the UK route-to-diagnosis literature, which has traditionally focused on the contrast between emergency and screening pathways [1,2,15].

Given its embedded role in triaging referrals [6,9], symptomatic FIT accounted for roughly half of the cohort. The proportion with advanced disease in this group was significantly lower than in incidental diagnoses. It was 8.9 percentage points lower than in emergency presentations, but this difference was not statistically significant (95% CI −1.1 to 19.0) and should not be taken to mean that the two routes have similar stage distributions. Emergency presentations were also heterogeneous. Obstruction or perforation accounted for 48/112 (42.9%) of presentations, while 51/112 (45.5%) presented with non-specific symptoms such as abdominal pain, loss of appetite or vomiting severe enough to prompt admission. In the UK, linked primary care data show that many patients later diagnosed as an emergency had prior GP contact, in some cases with documented red-flag symptoms [19,20]. Also, pre-existing comorbidity has been shown to delay colorectal cancer diagnosis in primary care [21]. However, presentation patterns alone do not demonstrate avoidable delay or missed referral opportunities. Whether emergency presentations in this cohort reflect delayed help-seeking, under-recognition of symptoms in primary care, rapidly progressive disease, or other factors cannot be determined from this dataset, and this warrants examination in future work.

Patients referred on symptoms alone had a proportion of advanced disease (79/131, 60.3%) almost identical to emergency presentations (68/112, 60.7%) and higher than symptomatic FIT (262/506, 51.8%), although these comparisons were not formally tested. This group may also differ from the FIT group in ways that affect stage, as some patients are referred without a FIT because features such as a rectal mass already make cancer likely [5,7]. Tumour location also differed by route. Right-sided tumours were proportionally most frequent in emergency (51/112, 45.5%) and incidental (23/53, 43.4%) diagnoses and least frequent in BSW (41/172, 23.8%). By contrast, rectal tumours were uncommon in emergency presentations (9/112, 8.0%) but formed about a third of symptomatic FIT (158/506, 31.2%) and symptoms-only (47/131, 35.9%) cases. This may reflect right-sided cancers being less likely to produce overt symptoms such as rectal bleeding and, within screening programmes, more likely to present as interval cancers [14]. However, symptoms by tumour site and site-specific FIT performance were not assessed here.

Only two patients were diagnosed after a positive FIT in the absence of CRC-related symptoms, and both had early-stage disease. Given the very small number, this group was described only, and no conclusions about this route can be drawn.

The principal value of this study lies in providing a contemporary, route-specific description of colorectal cancer diagnosis across a defined region. The findings identify where advanced-stage disease is most frequently encountered and how patient characteristics and presentation differ across diagnostic routes. This may provide a regional benchmark for future studies and help identify pathways where further investigation of the reasons for late-stage diagnosis is warranted.

Implications and future work

The main practical implication is that the pathways with the highest observed burden of advanced disease (incidental and emergency diagnoses and symptoms-only referral) are those where no FIT-based triage preceded diagnosis, although this study cannot show that FIT would have changed their stage. Future audit work could firstly fit a multivariable model for advanced stage including age, sex, tumour location and comorbidity. Secondly, link primary care records to examine prior consultations, FIT results, referral intervals and time to investigation, particularly for emergency presentations. Thirdly, record the route to diagnosis prospectively at MDT using a fixed hierarchy. Lastly, use a validated comorbidity index and analyse perforation and obstruction separately by stage.

Strengths and Limitations 

The main strengths of this study are its large cohort and its assessment of colorectal cancer diagnosis in routine clinical practice. Diagnostic routes were classified into six clinically distinct categories, allowing separation of pathways that are frequently combined in broader analyses, particularly symptomatic FIT from symptoms-only referral and incidental from emergency diagnosis. The study also examined stage distribution alongside presenting features, tumour location and the nature of emergency presentations, providing greater clinical context than route frequency alone. This also enabled identification of the high burden of advanced-stage disease within the incidental diagnosis group, a finding that may have been obscured by broader typical pathway classifications. 

Several limitations should be acknowledged. As a retrospective study using routinely collected clinical data, the analysis was dependent on the completeness and accuracy of existing documentation and was therefore susceptible to information bias. Route classification may also have been affected by misclassification where the distinction between symptoms-only, incidental, and emergency presentation was unclear from the available records. Stage was based on pre-treatment clinical/radiological staging where available and on pathological staging otherwise. Pathological staging was used more often in emergency presentations, where cancer was often identified at urgent surgery, and this may affect comparisons between routes. Patients without a determinable stage were excluded, which may have introduced selection bias, and some variables had missing data. Comorbidity was a crude binary variable (two or more conditions) rather than a validated index, and there was limited granularity in several variables; for example, symptoms were recorded as present/absent without duration or severity, and stage was not analysed separately for obstruction and perforation. The asymptomatic FIT group was too small to analyse. Also, the study includes only patients aged ≥ 50 years, limiting applicability to younger patients. Comparisons between diagnostic pathways were unadjusted for age, comorbidity, tumour location and other potential confounders, reflecting the descriptive nature of the study; therefore, residual confounding cannot be excluded. Pairwise testing was restricted to four pre-specified comparisons, and differences involving other pathways, including the symptoms-only group, were not formally assessed. Finally, the study reflects a single regional health board, and the findings may therefore not be directly generalisable to other settings.

## Conclusions

In this descriptive study of patients aged ≥ 50 years in one Welsh health board, route to diagnosis was associated with stage at presentation. In unadjusted analyses, which may partly reflect differences in patient characteristics and staging method, screening-detected cancers had the lowest proportion of stage III-IV disease and incidental diagnoses the highest. Approximately half of cancers diagnosed after a positive symptomatic FIT were already advanced; as only diagnosed patients were included, this does not assess the effectiveness of FIT. These findings provide a regional benchmark and identify pathways, particularly incidental and emergency diagnoses, where the reasons for late-stage diagnosis warrant further investigation.
